# Virus-Like Particle, Liposome, and Polymeric Particle-Based Vaccines against HIV-1

**DOI:** 10.3389/fimmu.2018.00345

**Published:** 2018-02-28

**Authors:** Yong Gao, Chanuka Wijewardhana, Jamie F. S. Mann

**Affiliations:** ^1^Department of Microbiology and Immunology, University of Western Ontario, London, ON, Canada

**Keywords:** HIV-1, vaccine, virus-like particles, nanoparticles, immunogenicity

## Abstract

It is acknowledged that vaccines remain the best hope for eliminating the HIV-1 epidemic. However, the failure to produce effective vaccine immunogens and the inability of conventional delivery strategies to elicit the desired immune responses remains a central theme and has ultimately led to a significant roadblock in HIV vaccine development. Consequently, significant efforts have been applied to generate novel vaccine antigens and delivery agents, which mimic viral structures for optimal immune induction. Here, we review the latest developments that have occurred in the nanoparticle vaccine field, with special emphasis on strategies that are being utilized to attain highly immunogenic, systemic, and mucosal anti-HIV humoral and cellular immune responses. This includes the design of novel immunogens, the central role of antigen-presenting cells, delivery routes, and biodistribution of nanoparticles to lymph nodes. In particular, we will focus on virus-like-particle formulations and their preclinical uses within the HIV prophylactic vaccine setting.

## Introduction

Human immunodeficiency virus-1 (HIV-1) and HIV-2 have emerged as the result of zoonotic lentiviral transmissions of simian immunodeficiency viruses (SIV), from chimpanzees/gorillas and sooty mangabeys, respectively, into the human population. First identified as the etiological agent behind acquired immunodeficiency syndrome (AIDS) in humans in the early 1980s, HIV has continued to spread into a global pandemic and major public health concern. At the end of 2015 alone, there were 36.7 million people infected with HIV with an additional 2.1 million new infections that same year. Excluding parenteral infections, HIV acquisition is almost entirely transmitted through sexual intercourse. Worldwide, approximately 80% of the 30 million people infected with HIV acquired the virus through heterosexual transmission, with receptive vaginal and anal intercourse accounting for 60–70 and 5–10%, respectively, of the global total.

Human immunodeficiency virus infection is characterized by the catastrophic depletion of CD4 T cells—the very cells that orchestrate host immune responses against invading pathogens. The combination of viral cytopathic effects, cell suicide by caspase-1 mediated pyroptosis, and caspase-3-mediated apoptosis are the major mechanisms behind the depletion of CD4 T cells (1). Over time, the continued depletion of CD4 T cells causes an AIDS-defining illness where people become susceptible to a plethora of opportunistic infections.

During heterosexual transmission, HIV-1 enters the body through the genital mucosa, while in male to male transmission, HIV-1 enters through the rectal or upper gastrointestinal mucosa. Numerous factors contribute to HIV-1 transmission rates across genital and gut mucosal surfaces, including the viral load in the donor during sexual contact, presence of pre-existing mucosal infections, trauma, inflammation, frequency of sexual contacts, male circumcision, receptive anal intercourse, and whether individuals used barrier contraceptives such as condoms. The current paradigm suggests HIV enters anatomical sites with micro-abrasions, likely occurring as a direct result of sexual activity, thus providing more direct access to target cells residing in submucosal tissues. It is important to note that mucosal frontline surfaces, such as the endocervix, transformation zone, and gastrointestinal mucosa, have only a single layer of columnar epithelium to separate the external environment from the sterile inner sanctum of the body. Thus, these surfaces are more susceptible to microtraumas and likely serve as entry points for HIV. After the initial seeding of an infecting founder population, the virus spreads to regional lymph nodes and then disseminates systemically. The narrow timeframe from breaching the mucosal frontline through to primary foci development and immediately prior to lymph node distribution is often referred to as the “window of opportunity” (2). This represents the instance where a potential vaccine candidate might be maximally effective at aborting an infection event. During this window of opportunity, HIV may also be at its most vulnerable state during infection considering only one or few HIV-1 clones were transmitted from a highly diverse HIV population in the recipient.

Due to the advent of antiretroviral therapies (ART), HIV is no longer a death sentence. From 1995–2010, approximately 5.1 million AIDS-related deaths were averted in low- and middle-income countries due to greater accessibility of ART (3). Current combination antiretroviral therapies (cART) inhibits various points of the viral replication cycle and, is most effective as a triple therapy regiment to prevent the induction of escape mutants. Although cART can decrease viral load in the blood to undetectable levels, it remains a non-curative strategy against HIV due to the presence of the latent reservoir (4). This is because cART can only target actively replicating virus and, therefore, cannot impact the latent reservoir.

## Early Attempts at HIV Vaccines

Initial HIV vaccine candidates to enter human clinical trials were subunit vaccines based on the surface Env glycoprotein. These were mainly soluble monomeric gp120 or gp160 constructs (5–7). These early attempts at vaccines provided the necessary evidence for protection against homologous SIV-HIV (SHIV) chimeric virus challenge in non-human primates (NHPs) (8). Unfortunately, these vaccines were not protective against a heterologous virus challenge despite inducing strong immune responses and the animals remained susceptible to infections with heterologous SHIV viruses (9). Despite this, numerous gp120 subunit vaccines have been designed and evaluated in human clinical trials to provide proof of principle for vaccine-mediated protection against infection. An example of an early monomeric gp120 vaccine trial conducted in human volunteers was the Phase III efficacy trial (VAX003). This study was conducted within an injection drug using cohort in Thailand. The vaccine (AIDSVAX B/E) consisted of a combination of recombinant clade B and E gp120 antigens adjuvanted with alum. The vaccine ultimately provided no significant levels of protection against infection among the participants, with 8.4% in the vaccine arm and 8.3% in the placebo arm becoming infected (10, 11). Another notable human vaccine trial was the VAX004, which employed the AIDSVAX B/B vaccine and showed near identical results in North America and in the Netherlands. This vaccine was given to men who have sex with men (MSM) and women at high risk for sexually transmitted HIV infection cohorts (12, 13). Despite eliciting high homologous neutralizing antibody titers against the applied vaccine strains, only weak neutralizing antibody responses were detected within recipients. Overall, both VAX003 and VAX004 vaccine trials failed to show protection from HIV infection, despite being immunogenic. Such studies helped initiate the search for new and improved immunogens and the development of oligomeric Env structures to better mimic the HIV-1 functional spike. In theory, trimeric Envs may be advantageous to predecessor monomeric vaccine candidates as it is thought they might be able to induce conformationally dependent antibodies. Most of the trimeric Env immunogens, the so-called gp140 immunogens consisting of gp120 and the ectodomain of gp41 (14–16), elicit significantly higher titers of neutralizing antibodies compared to the monomeric Env forms (17–21), however, the breath of neutralization is still limited. Attempts to enhance the immunogenicity of Env antigens, i.e., increase the neutralizing antibody levels, involved modifying Env molecules by deleting the highly glycosylated V2 domain. Unfortunately, this had minimal impact in eliciting broadly neutralizing antibodies. Trimeric gp140 with new disulfide bonds to stabilize gp120–gp41 interactions were tested for immunogenicity within novel prime-boost immunization protocols (22). When rabbits were primed with DNA encoding a membrane bound form of gp140 by electroporation followed by disulfide-stabilized trimeric gp140 booster immunizations *via* the intramuscular (IM), intradermal (I.D.), or subcutaneous (S.C.) routes, high titers of neutralizing antibodies against homologous viruses could be elicited.

Since these early failed attempts at vaccination, numerous DNA, and viral vector platforms have been created and evaluated as anti-HIV vaccine candidates. While DNA/RNA and viral vectors do not fall under the mandate of this review, it is worth mentioning the only clinical trial to date to have demonstrated any efficacy against HIV acquisition was RV144—the phase III “Thai Trial.” In 2009, the results from the RV144 trial were released, demonstrating the effectiveness of an ALVAC vaccine prime followed by an AIDSVAX boost. The ALVAC prophylactic vaccine component was a canarypox vector encoding HIV-1 Gag, Pol, and gp120 Env while the AIDSVAX boost consisted of gp120 B/E adsorbed to alum. A modest benefit of 31.2% vaccine efficacy was demonstrated in the vaccine arm using this regimen (23). Interestingly, in a *post hoc* analysis of the protection data, it was observed that should the study have stopped after the first year of implementation, vaccine efficacy would have been as high as 60%, indicating a non-sustained protection from acquisition was achieved. In terms of the immune correlates of protection a weak neutralizing antibody response was again observed; however, non-neutralizing plasma IgG binding antibodies to the top of the Env V1/V2 variable loop were associated with protection (24).

## Nanopharmaceutical-Based Vaccines

Soluble gp120 monomers and more advanced trimeric forms of Env failed to elicit the protective responses necessary to prevent HIV acquisition. Thus, a critical goal in HIV vaccine design has been to try and understand why this has been the case. Collectively, the knowledge that (1) infused neutralizing antibody protects against viral challenge in non-human primates (NHP) studies, (2) a percentage of individuals will generate neutralizing antibody responses against the autologous infecting virus, and that (3) highly potent neutralizing antibody can be generated in some individuals, solidifies the notion that an anti-HIV B cell response can be protective. As neutralizing antibodies are directed against the surface glycoprotein, presentation of natural Env structures, mimicking the native viral Env is deemed imperative to eliciting a neutralizing immune response. Resolving this scientific challenge has led to re-engineering of the Env itself to achieve a native conformation. As a result, numerous antigenic structures have been developed, including germline revertants targeting the N332 supersite and near native trimers candidates such as BG505 SOSIP.664 (25–27). These new immunogens, designed to harness anti-HIV neutralizing antibody responses can elicit autologous neutralizing antibody responses and depending on how they have been utilized, can elicit heterologous Tier 2 neutralizing antibody. However, these and other such candidate vaccine antigens do require mutations in their amino acid sequences to increase stability and this can come at the expense of losing antigenic conformation. For reviews more focused on HIV-1 Env-based vaccines, several recent articles have been published which may be of interest to readers (28, 29).

### Virus-Like Particles (VLPs)

To overcome these afore-mentioned issues in antigen design, Env trimers have been expressed in lipid membranes. This enables their conformations to more accurately resemble native and functional Env spikes found on infectious virus. Taking this into consideration, nanoparticle platforms such as VLPs provide the ideal technology to express HIV Env immunogens. VLPs are non-infectious and genomeless multiprotein structures that mimic the conformation of viral proteins in their natural environment. These self-assembling molecules can generate ordered arrays of polypeptides that come together to form the VLP. The resulting repetitive geometry offers maximal VLP–host interactions due to increased avidity (30). As a result, VLPs can present viral antigens in their authentic conformation and maximally stimulate resulting immune responses (31).

The use of VLPs as potential vaccine candidates offers numerous advantages over traditional strategies. VLPs have the unique ability to present viral Env spikes in their natural conformation and, therefore, the potential to elicit neutralizing antibody responses against HIV. This feature is often lost when antigens are purified from pathogens or when pathogens are rendered inert through chemical or heat inactivation. VLP vaccines are also cheaper than subunit vaccines since less of the vaccine can be administered without impairing the resulting immune response. Another attractive property of VLP vaccines are their particulate structure, which allows them to be efficiently taken up by antigen-presenting cells (APCs) and stimulate strong humoral and cellular immune responses (32, 33) since they can be presented on both MHC class I and class II molecules (34, 35). In addition, their polyvalency allows for efficient B cell receptor (BCR) crosslinking and activation.

Although several commercially available VLP vaccine formulations are available to protect against Hepatitis B, Hepatitis E, and human papillomavirus (HPV), none exist for HIV (Table 1). One of the prominent prophylactic VLP vaccine formulations against the Hepatitis B virus is RECOMBIVAX^®^. It mainly consists of Hepatitis B surface antigen that spontaneously assembles into ~20 nm lipid-containing particles (36). The major prophylactic vaccine against the hepatitis E virus is the Hecolin^®^ vaccine, whose major constituent is a small portion of the Hepatitis E capsid protein that self assembles into 20–30 nm particles (36). Gardasil^®^ is a major prophylactic VLP vaccine formulation against HPV. It is synthesized by expressing HPV L1 capsid protein, which spontaneously assembles into immunogenic non-infectious VLPs that can induce the same neutralizing antibody responses as the native virions (37).

**Table 1:** Pharmaceutical virus-like particle-based vaccines.

Name	Major antigen constituent	Approval status	Protection against	Approximate size	Route of immunization	Adjuvant used
Recombivax™	Hepatitis B surface antigen with Hepatitis B-derived lipids	First approved in USA, 1983	Hepatitis B	20 nm	IM	Alum

Hecolin^®^	Hepatitis E capsid protein	First approved in China, 2011	Hepatitis E	20–30 nm	IM	Aluminum hydroxide

Gardasil™	Human Papillomavirus (HPV) L1 capsid protein	First approved in USA, 2006	HPV 6, 11, 16, 18	55 nm	IM	Alum

Cervarix^®^	HPV L1 capsid protein	First approved in USA, 2009	HPV 16, 18	55 nm	IM	AS04

*IM, intramuscular*.

There are five main platforms that are currently utilized to produce VLP vaccines: bacteria, yeast, insect cells, mammalian cells, and plants. Although leveraging bacteria to produce VLPs is the most scalable and cost-effective approach, they are unable to perform the essential post-translational modifications for optimal immunogenicity in human (31). In addition, the use of bacteria may introduce contaminating endotoxins into vaccine formulations. Yeast are also scalable and cost-effective approaches to producing VLPs; however, unlike bacteria, they can perform eukaryotic post-translational modifications such as glycosylation. Insect cells can be induced to produce VLPs through baculovirus expression systems. This system can produce large quantities of VLPs in their natural conformation but must be purified from baculovirus contaminants. In fact, these components may obscure the immune response against the epitopes of the VLP (38). VLP production through mammalian cells cannot be scaled up as efficiently as bacteria, yeast, or insect expression systems. In addition, the use of mammalian cell systems runs the risk of human viral pathogen contamination (39); however, they have the most accurate and complex post-translational modifications (40). Therefore, this system is particularly advantageous for constructing complex VLPs. Plants can be induced to produce VLPs through *Agrobacterium tumefaciens*, a Gram-negative soil-based bacteria used to transform plant cells (41). Although plant-based expression systems are easy to scale up and contain no human-derived viral contamination, these VLPs cannot undergo the same post-translational modifications that are done in mammalian cells, thus reducing immunogenicity.

The HIV-1 Env trimer is synthesized as a 160 kDa precursor and is processed by protease to yield a surface and transmembrane subunit during its passage through the secretory pathway. The resulting surface bound oligomer comprises three non-covalently associated gp120 and gp41 subunits. The surface gp120 is responsible for binding host cell target receptors (CD4) and co-receptors (CCR5) whereas the gp41 anchors Env in the membrane. Taking the well-characterized influenza virus as a model system shows that the virus may require only 2–3 trimers to engage with its cellular receptors to create a pre-fusion structure. However, it is believed that 6–8 trimers may be necessary to build the stable fusion pore (42). This question has been hard to address in the context of HIV-1 for a number of reasons including the fact that (1) a large percentage of HIV virions (>99%) are replication defective, (2) the sparsity of intact Env trimers on the viral surface (7–14 spikes/virion), (3) spontaneous shedding of gp120 from Env complexes, and (4) heterogeneity among the HIV-1 Env complexes (42). This has resulted in a number of conflicting studies suggesting a single trimer is necessary (42) or perhaps between five and eight trimers may be necessary for infection (43, 44). More recently, work done by the Trkola group has suggested divergent HIV strains differ in their stoichiometry for entry, requiring ~1–7 trimers, with the majority of HIV strains requiring 2–3 trimers for infection (45). In the context of eliciting humoral immune responses within the host, the surface Env density is important for efficient BCR crosslinking, leading to B cell clonal expansion and antibody affinity maturation. Unfortunately, HIV’s low density of Env spikes (7–14 spikes/virus) is substantially lower than that seen for other viruses, such as influenza (400–500 spikes/virus), vesicular stomatitis virus (VSV, 1,200 spikes/virion), Rous sarcoma virus (RSV, ~118 spikes/virion), and even compared to the related SIV (~70 spikes/virion) (46). Therefore, attempts to increase surface HIV Env density on VLPs may facilitate more protective immune responses. Using fluorescence activated cell (FACS) sorting to isolate producer cells that are recognized by trimer cross reactive broadly neutralizing antibody, Stano et al., sought to isolate and propagate producer cells displaying higher levels of antigenically correct Env (46). Following multiple rounds of FACS sorting, monodisperse VLP formulations were generated that had a 10-fold higher Env density per particle (~127 spikes/particle) than control viral particles. When the Env was sequenced, a truncation in the C-terminal tail of Env was seen. Although the high-density Env particles displayed a greater infectivity compared to normal pseudoviruses, they exhibited similar levels of neutralization sensitivity (46). However, the high Env density VLPs were superior activators of broadly neutralizing VRC01 B cells as determined by upregulation in CD69, IL-6, and TNF-a production and BCR downregulation. This collectively indicates superior BCR clustering and activation (46).

One of the major roadblocks to an effective HIV-1 vaccine is the sheer diversity of the viral swarm. A preventative vaccine must be protective against HIV strains/subtypes circulating in multiple geographic regions. Polyvalent Env vaccines are one such approach to overcome the viral diversity and involve the use of heterogeneous Env mixtures to expose developing immune responses to a multitude of Env conformations (47). This is thought to help promote immuno-focusing of B cell responses onto more conserved regions of vulnerability such as the Env CD4-binding site and MPER while also promoting antibody breadth. To address this issue, Pankrac et al. developed a chimeric HIV-1 VLP formulation capable of accommodating near full length (NFL) genomes of HIV-1 that captures the viral diversity within infected individuals (47). To do this, the authors took the plasma of five pre-cART HIV positive volunteers and engineered the isolated virus into a mixed VLP formulation. By inserting mutations into integrase and a series of substitutions into the RNA packaging element, the resulting VLPs had dramatically reduced genomic RNA and lost the functional ability of viral integrase. They also deleted the 5′LTR so that the probability of reverse transcription was eliminated. Collectively, these “dead” viral particles were shown to have similar morphology to wild-type virus, contained p24 and p17, as well as expressed functional Env, Tat and Rev (47, 48). Upon exposure of HIV-1 infected CD4 T cells to dendritic cells (DCs) pulsed with the heterologous VLPs, a strong IFN-g response was detected, indicating the ability to generate antigen-specific CD4 T cell recall responses. In addition, when the VLPs were used to pulse PBMC cultures, the authors demonstrated Granzyme B production, a biological marker for cytotoxicity (47). Finally, the VLPs were also shown to be able to prime and boost a CD4 adaptive immune response *in vitro* using healthy donor CD4 T cell-DC co-cultures (47). This polyvalent VLP vaccine formulation is called ACT-VEC and is now being evaluated in non-human primates (NHPs) for its immunogenicity.

Trying to understand the host and viral factors that influence HIV infection and how the virus crosses the mucosal barrier might lead to more efficacious HIV vaccines. This has led to the characterization of the phenotypic properties of transmitted/founder (T/F) viruses to see if transmission is through stochastic events or whether the genetic bottle neck selects for viruses with certain properties. Features associated with transmitted viruses include CCR5 tropism, short variable loops and less N-linked glycan residues. With such distinguishing features in mind, McClure et al. tested the antigenicity and immunogenicity of DNA and modified vaccinia Ankara expressed VLPs, displaying native forms of T/F clade C Envs (49). The hypothesis being that the VLPs expressed Envs with or without administered gp120 might elicit a broadly neutralizing antibody response. This interesting study proceeded investigations into the clade C infection occurring within an infected individual that eventually developed a broadly neutralizing antibody response against the CH103 CD4-binding site (50). Significantly, the VLP expressing vectored immunogens boosted with gp120 were immunogenic in NHPs and despite a complex regimen elicited a neutralizing antibody response in 50% of the macaques tested, with antibody directed toward the CD4-binding site. Thus, demonstrating that a VLP prime plus gp120 boost using a T/F Env to be capable of inducing autologous Tier 2 neutralizing antibody to the CD4-binding site (49).

### Liposomes

Antigen delivery by liposomal vesicles was demonstrated over 30 years ago when unilamellar vesicles were generated that were composed of phosphatidylserine. These liposomes contained poliovirus and purified poliovirus RNA. They were found to be infectious and could be potentially utilized as a delivery agent for biological macromolecules (51). Liposomes can either be microparticles or colloidal carriers (52), with a diameter ranging from 20 nm to more than 10 µm (53). There are many types of liposomes (e.g., multilamellar vesicles and large or small unilamellar vesicles), with the conventional ones composed of biodegradable and non-toxic zwitterionic phospholipids, such as phosphatidylcholine, phosphatidylserine, and cholesterol (52–54). The formation of liposomes is a spontaneous occurrence when lipids are subjected to hydration (52), enabling the formation of lipid bilayers surrounding an aqueous core. The first efforts at using liposomal technologies for delivery of candidate HIV vaccine antigens occurred nearly 25 years ago by Bui et al. (55). In this first attempt, the authors utilized a non-glycosylated, denatured gp120 called HIV Env-2-3-SF2 as the vaccine immunogen, and co-formulated the antigen with either alum or liposome. The HIV Env-2-3-SF2 alum and HIV Env-2-3-SF2 liposome immune responses were then potentiated using liposomal IL-7. The result was the demonstration that liposomal HIV Env antigens elicited stronger antibody titers than the alum formulations and that the responses could be enhanced using liposomal IL-7. Notably, cytotoxic T lymphocyte (CTL) responses were greater in the liposomal arm than the alum treatment regimen (55).

Interbilayer-cross-linked multilamellar vesicles (ICMV) with His-tagged gp140 trimers anchored onto the surface of the delivery agent using Ni-NTA functionalized lipids were recently explored (56). The aim being to deliver a high quality, B cell triggering-gp140 trimer immunogen, previously demonstrated to elicit high titers of potent cross clad neutralizing antibody responses (57). The ICMV-gp140 nanoparticles were adjuvanted with TLR4 agonist MPLA and used to assess humoral immune response induction in mice and compared to gp140 adjuvanted with SAS (oil in water adjuvant containing MPLA trehalose-6,6′-dimycolate). The trimer-loaded ICMV particles were superior inducers of humoral immune responses compared to gp140 alone or adjuvanted with SAS, with the breadth of the anti-HIV antibody response increased in response to ICVM surface anchoring—including against the conserved MPER sequence of gp41. Unfortunately, due to the use of the murine model, the authors did not test neutralizing antibody production (56).

In a separate study, Ingale et al., utilized single bilayer liposomes, displaying well-ordered, high-density trimers, such as JRFL-SOSIP, and JRFL-NFL, for better B cell activation (58). The trimers were conjugated to the surface of the liposomes, by electrostatic interaction using the cholesterol substitute, DGS-NTA(Ni), and c-terminal His-tagged HIV-1 trimers. As a result, the liposomal formulation containing the surface trimers more efficiently stimulated B cell germinal center formation compared to soluble trimers demonstrating the necessity for membrane display. Furthermore, there appeared to be a suggestion that the liposomal trimers promoted stronger antibody titers to the native trimers and to generate low level tier 2 homologous neutralizing antibody (58). As antigens coupled to the surface of liposomes are exposed to interstitial lymphatic fluid as they transit into the draining lymph nodes, the Bale et al.’s study suggested that it might be possible for surface displayed antigen that are non-covalently bound to be susceptible to dissociation from the liposome prior to B cell contact (59). Therefore, the authors created a second-generation liposomal formulation where HIV-1 Env trimers were coupled to synthetic single layer liposomes by alternative means. Here, the authors found that maleimide-thiol covalent coupling of trimers to liposomes elicited higher anti-HIV Env IgG antibody responses than soluble trimers or trimers coupled to liposomes *via* non-covalent metal chelation. Furthermore, the liposomes-coupled antigens were better activators of B cells as determined through Ca^2+^ flux measurements. Finally, murine immunogenicity studies demonstrate that the covalent coupling of antigens enabled the expansion of B cell germinal center reactions, over and above what was seen with soluble antigens, and that they also blocked the access of B cells and antibody to the c-terminal end of the exposed Env spike, suggesting that the trimers were intact at the point of immune recognition and adaptive immune activation (59). Taken together, this suggest that the particulate display of high-density antigen arrays on the surface of liposomes elicits improved B cell responses over previous soluble versions of the same trimers.

A promising vaccine candidate has been the membrane proximal region (MPER) of Env. This gp41 region is the target for several neutralizing antibodies and is attractive as a vaccine candidate due to its conserved and linear nature. A significant drawback to MPER immunogens is its inherent lack of immunogenicity. Recently Hanson et al. utilized 150 nm liposomes as membrane display vehicles to promote a more native vaccine antigen conformation (60). In this example, the authors evaluated the strength and durability of humoral immune responses elicited by MPER peptides anchored to the surface of liposomes *via* palmitoyl tails. The liposomal anchored peptides were compared to alum and complete Freund’s oil-based emulsion adjuvant. Liposomal anchored peptides successfully elicited MPER-specific IgG antibody while alum and Freund’s adjuvants did not. Critically, anti-MPER antibody responses could be augmented through the inclusion of adjuvants, such as TLR4 agonist (MPLA), TLR9, or STING Interestingly, intrastructural help through helper epitopes promoted IgG responses to MPER without increasing B cell responses against the help sequence (60).

### Polymeric Nanoparticles

Polymeric nanoparticles are very promising candidate delivery systems and adjuvants for a range of vaccine antigens. Their favorability stems from their ease of synthesis, biocompatibility, their biodegradable nature, the fact they are non-immunogenic, non-toxic and fairly inexpensive. Numerous different polymeric nanoparticle exist but the most frequently encountered types include chitosan, poly(lactic-co-glycolic acid) (PLGA) and poly(lactic acid) (PLA) (Table 2).

**Table 2:** (A) Liposome and polymeric particle vaccines; (B) liposome and polymeric particle vaccines against human immunodeficiency virus (HIV)/simian immunodeficiency virus (SIV).

Particle type	Antigen arrangement	Resulting immune response	Adjuvant used	Animal model	Publication
**(A) Liposome and polymeric particle vaccines**					
Liposome	ICMV with His-tagged gp140 trimers anchored onto Ni-NTA functionalized liposome particles	Humoral immune response induced. Breadth of anti-HIV responses increased in response to ICVM surface anchoring	MPLA	Mouse	(56)

Liposome	Single bilayer liposomes displaying high-density His-tagged JRFL-SOSIP and JRFL-NFL Env trimers. Conjugation to liposomal surfaces through DGS-NTA(Ni) non-covalent linkage	B cell germinal center formation and induction of low-level tier two homologous antibodies	ISCO-MATRIX	Mouse	(58)

Liposome	CLDCs are particles loaded with antigen and DNA containing CpG ODN motifs. The cationic component of CLDCs ensure entry into endosomal compartments, whereas the CpG ODNs trigger endosomal TLR9	Animals immunized with CLDC adjuvanted SIV-derived antigens developed more robust SIV-specific T and B cell responses compared to animals that were not immunized with CLDC. In addition, CLDC-treated animals developed better memory response as evident following immunization with whole AT-2 inactivated SIVmac239	CLDC	Rhesus macaque	(61)

Liposome	Single bilayer liposomes displaying high-density His-tagged Env trimers conjugated to liposomal surfaces through maleimide-thiol covalent linkage	Anti-HIV Env IgG responses elicted. Increased activation of B cells and germinal center formation	ISCO-MATRIX	Mouse	(59)

Liposome	MPER peptides anchored to liposomes surface through palmitoyl tails to form 150 nm particles	Induction of anti-MPER antibody responses that is maximized by adjuvanting with MPLA, or TLR9, or STING agonists	Alum, Freund’s adjuvant, MPLA, TLR9, and STING agonists	Mouse	(60)

**(B) Liposome and polymeric particle vaccines against HIV/SIV**					

Chitosan	HIV-1 clade C gp140 co-formulated with chitosan and delivered intranasally	Induction of CD4 T cell responses. Serum antibody responses were generated following IM boost	Chitosan	Human	(62)

Chitosan	Trimeric CN54gp140 co-formulated with chitosan	Increase in systemic IgA and IgG anti-gp140 antibodies following intranasal and sublingual delivery	Chitosan	Mouse	(63)

PLGA	HIV-1 p24-Nef peptide chemically conjugated to TLR5 agonist, FLiC and co-formulated with PLGA nanoparticles	Increased IgG1 and IgG2a titers, cytotoxic T lymphocyte (CTL) killing activity, and lymphocyte proliferative response following ID immunization	FLiC	Mouse	(64)

PLGA	PLGA encapsulated TLR9 agonist, CpG, and MPLA with HIV CTL epitopes	Strong immune response against multiple splenocyte CTL epitopes as measured by IFN-g release	MPLA and CpG	C57BL/6 mouse	(65)

*ICMV, interbilayer-cross-linked multilamellar vesicles; Ni-NTA, nickel-nitrotriacetic acid; MPLA, 3-*O*-deacyl-4′-monophosphoryl lipid A; SAS, Sigma Adjuvant System; PLGA, poly(d,l-lactic-co-glycolic acid); CLDC, cationic liposome-DNA complex*.

#### Chitosan (Poly(d-Glucosamine))

This cationic polysaccharide, polymeric nanoparticle is derived from the deacetylation of chitin, a naturally occurring polymer found in the cuticles of insect species and crustaceans, such as crabs and shrimp. Due to its strong immune-stimulatory properties and low immunogenicity, chitosan has frequently been utilized as an antigen delivery system. A significant advantage of chitosan to conventional adjuvants such as alum is its ability to promote T_H_1 immune responses and to also act as both delivery system and adjuvant when applied to mucosal surfaces.

In 2016, Cosgrove et al., published the first comparative Phase 1 clinical trial investigating the safety and immunogenicity of three HIV-1 clade C gp140 vaccinations delivered by either the IM, intranasal (IN), and intravaginal routes in women (62). The vaccine antigens were co-formulated with either glucopyranosyl lipid adjuvant (GLA), chitosan nanoparticles or an aqueous gel, respectively. Within this study, chitosan was ineffectual at promoting humoral immune responses despite the evidence from previous clinical studies, suggesting that chitosan increased potent antibody responses to vaccine immunogens. Interestingly, the authors noted that gp140 co-formulated with chitosan and intranasally delivered resulted in induction of CD4 T cell responses and upon IM boosting, all individuals generated a detectable serum antibody response. This suggests, that the chitosan-gp140 mucosal vaccination primed for an anamnestic serological response. However, it is unclear as to why the priming was restricted to CD4 T cells or if it had indeed primed B cells, but the responses were too low to measure (62). How nanoparticulate chitosan acts as adjuvant is currently unknown; however, recent evidence suggests that it promotes DC maturation through type 1 interferons and increases antigen-specific T cell responses in a type 1 IFN receptor-dependent manner (66). The latter requiring cytoplasmic sensors cGAS and STING, as well as both mitochondrial reactive oxygen intermediates and cytoplasmic DNA (66).

Chitosan has also served as a formulation adjuvant by promoting penetration enhancement of formulations, enabling their uptake and increasing the bioavailability of vaccine antigens when applied topically to mucosal surfaces. This was clearly demonstrated by Klein et al., who compared various polymeric penetration enhancers to promote trans-mucosal delivery of trimeric CN54gp140 protein (63). In this study, chitosan increased the systemic antibody levels of both IgG and IgA anti-gp140 antibodies following IN and sublingual delivery and increased antigen-specific antibody responses in the vagina of intranasally vaccinated mice. It is clear in this study that various polymeric formulations had differing abilities to augment immune responses and that the route of delivery was also a significant factor (63).

#### PLGA (Poly(d,l-Lactic-co-Glycolic Acid)) Nanoparticles

The use of PLGA as a nanoparticle vaccine delivery system has been studied comprehensively over the last two decades, resulting in numerous publications. However, it is the ability of PLGA nanoparticles to entrap bacterial toxoids/antigen or surface display antigens and induce long-lasting immune responses in small animal models which makes them an attractive delivery system (67–71). Early attempts at using PLGA formulations for delivery of recombinant HIV gp120 were centered on the ability of PLGA to release antigens over an extended period, promoting continuous antigenic exposure and immune education (72). These PLGA vaccines were designed to be single shot vaccines, which provide a pulsatile release of contained antigen and QS-21 adjuvant at intervals of 6 months. The resulting antibody response caused neutralization against the matched strain of HIV-1 and providing evidence for PLGA to be a slow release system (72). Since these early attempts, sophisticated attempts at using PLGA formulations as an HIV vaccine have been made (Table 2). For instance, the HIV-1 p24-Nef peptide was chemically conjugated to TLR5 agonist FLiC and co-formulated with PLGA nanoparticles (64). Upon intradermal vaccination in mice, the combination of TLR agonist and nanoparticle was shown to increase vaccine immunogenicity by increasing lymphocyte proliferative responses reducing the immunogenic dose required (64). In another example, Rubsamen et al. utilized PLGA encapsulated TLR9 agonist, CpG, and TLR4 agonist Monophosphoryl lipid A (MPLA) in the injection solution to augment immune responses to the delivered HIV CTL epitopes (65). The formulations resulted in immune responses against multiple splenocyte CTL epitopes as measured by IFN-g release (65).

## Nano-Vaccine Trafficking and Interactions with Host Immune Cells

The immune response to nanoparticle formulations such as liposomes, poly(d,l-lactide-co-glycolide) (PLGA), and VLPs are known to elicit both arms of the immune response. Understanding the cellular events involved in such vaccines are critical for their development as vaccine strategies and advancing test formulations to human clinical trials. Specifically, the molecular and cellular interactions occurring between nanoparticles and the vaccinated host immune system (i.e., effects on DCs, T and B lymphocytes, and lymphoid tissues) will be essential. Although many alternative vaccination routes have been explored in animal models and in humans, most vaccine studies employ IM vaccination. Intradermal (ID) vaccination demonstrates strong immune reactions to vaccinating antigens in a dose sparing manner compared to IM vaccination; however, ID vaccination is associated with a higher frequency of undesired side effects. Thus, we will concentrate on nanoparticle vaccines administered *via* the IM route.

The skeletal muscle system is one of the largest cellular compartments within the human body. Muscles are composed of large multinucleated syncytial muscle fibers which generate the necessary mechanical forces for locomotion. Following an injury to the muscle fibers, the surrounding satellite cells become activated and proliferate. The resulting myeloblasts fuse to become multinucleated myotubes, which will then go on to differentiate into mature muscle fibers. Under non-inflamed conditions, only a very few resident immune cells have been described to be present in the muscle (73). These include APCs such as macrophages and DCs; however, during pathological conditions, other immune cells such as monocytes, neutrophils, and lymphocytes can infiltrate the tissue (74). The introduction of vaccine antigens such as nanoparticles with adjuvants into the muscle environment induce transient inflammatory reactions at the delivery site, resulting in immune cell infiltration (74). Muscle cells can respond to the inflammatory mediators that are secreted by neighboring myocytes as well as resident and infiltrating immune cells. Myocytes express a range of receptors for cytokines, such as IL-1, IL-6, and IFN-g. Furthermore, they express chemokine receptors, such as CCR2, CCR4, and CCR10, which enable them to participate in surveying the local environment (75). The introduction of nanoparticles into the musculature enables their uptake by APCs such as DC and macrophages that are in the epimysium and perimysium interstitial spaces surrounding the entire muscle and muscle fascicles (76). The APCs produce pro-inflammatory mediators and chemokines that activate the myocytes and attract circulating immune cells and migrating APCs into the tissue. In support of this pro-inflammatory role of myocytes within the vaccinated muscle was the research published by Mosca et al. They used microarray and immunofluorescence analysis of the murine muscle after oil-in-water emulsion MF59 adjuvant, CpG, or alum injection (77). While CpG and alum induced time-dependent changes in 387 and 312 genes, respectively, MF59 induced changes in 891 genes, with recruitment of MHC II and CD11b cells within the injection site. Interestingly, the early response proteins penetraxin 3 and JunB were induced upon MF59 delivery, indicating that MF59 directly activated muscle fibers (77). Furthermore, IM injection was shown to be associated with release of ATP from local muscle fibers, which was important in the recruitment of immune cells to site of vaccination and the magnitude of the resulting immune response (78).

Neutrophils and monocytes have been found to be the first cells to infiltrate vaccinated muscle. This infiltration is normally detected within 3–6 h post-delivery. DCs are essential to adaptive immune response, and it is, therefore, not surprising that their recruitment is also rapid. These APCs take up exogenous nanoparticles, become activated, and in the case of DCs, migrate toward the draining lymph nodes. During this transit, the DCs upregulate co-stimulatory molecules, such as MHC II, CD80/86, and CD40. They also secrete cytokines and lose the ability to phagocytose antigens in preference for increased presenting capabilities. Once in the draining lymph nodes, the DCs initiate the adaptive immune response through activation of T and B cells. Remarkably, it is now becoming apparent that upon vaccination, intact nanoparticles such as VLPs can enter the draining lymph node without the requirement for DC-mediated transport (79). Using a mouse model, Cubas et al., demonstrated that IM vaccination with SHIV VLPs efficiently trafficked to the draining lymph nodes, although some speculation arose regarding VLP nanoparticle uptake by blood capillaries in the muscle and trafficking to the spleen (79). Nevertheless, once the VLPs were in the lymph node, the intact SHIV VLPs were detected in the subcapsular sinus (79). Once antigens enter the lymph node, they have been shown to reside there for extended periods of time. In the case of HIV-1, follicular DCs have been shown to retain non-degraded, viral particles in the form of immune complexes on their dendrites for months (80), thereby promoting germinal center reactions; however, it is unclear how long the SHIV VLPs were detectable in this instance.

Many factors, including, size, shape, charge, and receptor–ligand binding potential of nanoparticles dictate their distribution and cellular uptake upon vaccination, and therefore impact prevailing immune responses. VLPs are typically 20–150 nm in diameter, and excluding charge and receptor tropism, the size of the nanoparticle plays an important role in uptake and immunity. Generally, speaking nanoparticles and VLPs between 25 and 40 nm can effectively penetrate tissues upon delivery (81) and must exceed 10 nm to escape renal filtration. Nanoparticle vaccines exceeding 500 nm are likely taken up by APC at the injection site while nanoparticles and VLPs below 200 nm are mainly internalized by DCs, and in the free, intact, form will more likely accumulate in the liver and secondary lymphoid structures (82).

Over the years, numerous nano-vaccine delivery technologies have been investigated for optimal immune priming by examining the size of the nano-formulation (83–85). Using a combination of electron microscopy and dynamic light scattering, Mann et al., revealed that orally delivered, low diameter lipid-based vesicles (10–100 nm), containing influenza vaccine antigen, elicited a T_H_2 biased immune response in mice. This contrasted with similar vesicle compositions with an average diameter of 980 nm, which had a significantly greater T_H_1 bias (86). This work was supported by other previous research using lipid-based nanoparticle formulations delivered parenterally, where small lipid-based nano-vaccines (<155 nm) promoted T_H_2 responses while larger vesicles (>225 nm) elicited T_H_1 biased responses (87). It has been suggested that the T_H_1/T_H_2 polarizing effects related to nano-vaccine size might be due to efficiency of antigen presentation by the phagocytosed nano-vaccines by the APCs. An interesting manuscript by Brewer et al. provided evidence for altered trafficking of internalized antigens dependent on delivery systems size. Using murine macrophages, the authors described the trafficking of antigen loaded, large particles (560 nm), into early endosome-like phagosomes, whereas the smaller particles (155 nm) and soluble antigen were rapidly localized to endo-lysosomes (88). In this case, MHC II was detected in both compartments regardless of the T_H_1/T_H_2 bias. The internalization of large vesicles was also found to be mediated by actin-dependent phagocytosis, while the smaller vesicles were taken up in an actin-independent manner (88). These findings taken together suggest that nanoparticle size could significantly influence not only vaccine antigen uptake by APCs, but also influence their intracellular trafficking potential, dissemination around the body, and the ultimate immune outcome.

## Strategies to Increase Immunogenicity of Nano-Vaccines

One of the basic tenants of immunology states that antigens can induce immune responses, if it is perceived as being “foreign” by the host. However, non-self-antigens do not always trigger immune responses. Even if they do, they can be poorly immunogenic. For the immune system to become sufficiently mobilized, it must identify some sort of “danger.” This can occur in a number of ways including, (1) the recognition of exogenous signals by innate immune cells through pathogen-associated molecular patterns (PAMPS) and (2) being activated in response to recognition of danger-associated molecular patterns, which are endogenous molecules released by damaged or perturbed tissues/cells (89). Such immune stimulatory signals are often applied to vaccines in the form of adjuvants (90–92). Adjuvants are defined as agents which act to enhance the quantity and quality of immune responses to co-formulated immunogens. Mechanistically, adjuvants work by either simply arranging, aggregating, transporting, shielding antigens, or by stimulating pro-inflammatory signaling pathways conducive to enhancing immunogenicity. In other words, adjuvants increase the strength and effectiveness of vaccines. As an example, live attenuated vaccines do not necessarily require any additional help in generating immune responses as they exhibit low level infections. In contrast, highly purified subunit and inactivated vaccines are often poorly immunogenic and may not sufficiently activate innate immunity and generate the necessary pro-inflammatory environment that is conducive to the production of protective responses. Nanoparticle formulations such as VLPs may also display poor immunogenicity, compared to live attenuated vaccines and are therefore often delivered with an adjuvant to maximize an efficient immune response. Over the years, numerous adjuvant technologies have been applied to nanoparticle formulations. In an attempt to attain more and more protective responses and to also overcome potential side effects associated with highly immune-stimulatory adjuvants, mixed adjuvant regimens have gained in popularity. Recently, a number of excellent reviews have been published concerning adjuvants for HIV-1 vaccines (93, 94), so we will discuss only a few of the more common adjuvants below, some of which are detailed in Table 3.

**Table 3:** Adjuvants used in HIV vaccine strategies.

Name	Structure	Proposed mechanism of action
Alum	Polydisperse crystalline particles that are 2–10 µm in diameter	Antigen depot effect at vaccination site (95) in addition to potent IL-1 secretion and NLRP3 inflammasome activation (96)

MPLA	LPS-derived MPL molecules lacking: O-antigen, a fatty acid chain, and a phosphate group	Promotes T_H_1 immune responses without the safety concerns that are associated with LPS

GLA	Synthetic lipid A-like molecule administered in AF or SE formulations	TLR4 agonist that promotes T-bet-dependent T_H_1 immune response in addition to enhancing protection against a range of intracellular pathogens (97). GLA adjuvant effects are MyD88- and TRIF dependent

Flagellin	Major protein constituent of Gram-negative flagella. Usually incorporated into VLP vaccines	TLR5 agonist that is dependent on MyD88. It activates NF-κB in epithelial cells and APC populations (98)

AS04	Combination adjuvant that consists of alum and MPLA	Combining alum and MPLA causes a synergistic effect and results in the induction of higher quality antibody and neutralizing antibody titers (99)

MF59	Squalene-based	Induces production of antigen-specific CD4 T cell responses in addition to robust memory T and B cell responses

ISCOMATRIX	A matrix of saponin, cholesterol, phospholipid, and hydrophobic antigens which form molecular cages that are 40–50 nm in diameter (100)	Potent inducer of T_H_1 and T_H_2 responses which results in high frequency antigen-specific CD8 T cell responses (100)

Hiltonol	Poly-IC derivative: synthetic dsRNA that is stabilized using poly-lysine	TLR3 agonist that promotes the production of DC1 (101)

Resiquimod	Low molecular weight tricyclic molecule	TLR7/8 agonist that is dependent on MyD88

CpG DNA	Single-stranded DNA molecule containing multiple CpG motifs	TLR9 agonist that promotes the induction of a T_H_1 response

*This list is not exhaustive; it covers the more commonly used adjuvants. AS01 [used in HIV vaccines (102)], AS02, AS03, and CAF01 [used in HIV vaccines (16, 103)] among others are not on the list*.

*LPS, lipopolysaccharide; GLA, glucopyranosyl lipid adjuvant; AF, aqueous nanosuspension; SE, squalene-based oil-in-water nanoemulsion; Poly-IC, polyinosinic–polycytidylic acid; DC1, type 1-polarized dendritic cells*.

### Alum

Alum is one of the first adjuvants to be developed for augmenting vaccine elicited immune responses having first been demonstrated to act as an adjuvant by Glenny et al., in 1926. Since then, aluminum adjuvants have become the most widely utilized adjuvant and have been used in diphtheria, tetanus, pertussis and poliomyelitis vaccines in many countries for more than 70 years. Alum salts, form polydisperse crystalline particles (~2–10 μm in diameter) capable of adsorbing subunit vaccines onto their surface, thereby increasing BCR crosslinking, presentation to APCs, and can act to form an antigen depot at the vaccination site (95). Critically, alum adjuvants have been described to be potent inducers of IL-1 secretion *in vitro* within APC population, such as DCs and macrophages via NLRP3 inflammasome activation (96). While Alum has been used as an adjuvant in licensed HPV, HBV, and HEV VLP vaccines, how it functions as an adjuvant in the context of VLP vaccines is not clearly understood. Certainly, VLPs are readily phagocytosed by APCs due to their particulate nature. Furthermore, they are decorated with vaccine antigens on their surface, which enables BCR crosslinking. Thus, the adjuvant effects of alum, in the context of these VLP vaccines, may be due to antigenic depot effects and activation of the inflammasome.

The use of alum to augment antiviral immunity has been evaluated in the context of therapeutic HIV vaccines. Although therapeutic vaccinations are now aimed at a sterilizing or functional cure, initial attempts in the 1990s and early 2000s were made to therapeutically “modulate” immune responses to curb CD4 T cell decline and lower the rate of AIDS progression (48, 104). As decreasing anti-p24 responses had been correlated with clinical progression to AIDS, a p24-based vaccination was developed as a potential method to elevate p24 immune responses in HIV-infected individuals. In a randomized placebo-controlled trial that enrolled 304 HIV-infected individuals (CD4 counts <350 cells/μl), individuals were administered monthly IM injections of either (1) alum, (2) p24 VLP (500 μg) + alum, or (3) p24 VLP (1,000 μg) + alum (105). The volunteers maintained ART during the study which consisted of Zidovudine (ZDU) with or without didanosine or zalcitabine. CD4 T cells counts were monitored for the 6 months of treatment and then for an additional 6 months of follow-up. The study failed to demonstrate any benefit of immunization with p24 VLP despite adjuvanting with alum. Interestingly, no statistically significant antibody responses were detected against p24 or p17 but instead were detected against the carrier protein, Ty. Furthermore, a decline in CD4 T cells was recorded over time with no statistically significant difference between treatment regimens (105). In a similar follow-up study, 61 individuals were recruited and split into three treatment arms and received either (1) ZDU + IM alum, (2) ZDU + IM p24 VLP (500 μg) + alum, or (3) placebo capsules + IM p24 VLP (500 μg) + alum (106). This was conducted to evaluate the therapeutic effect of p24 VLP vaccination with ZDU on p24 antibody production and CD4 T cell counts, as well as any impact on viral loads. Despite the treatment being well tolerated, no significant differences between the treatment arms were recorded in any of the antibody and cellular immune responses measured (106).

Alum has also been used as an adjuvant for VLPs used for prophylactic vaccine strategies for prevention of HIV acquisition. Insect cell expression systems have been used to generate chimeric VLPs that express the V3 domain and a linear section of the discontinuous CD4-binding domain of gp120 within gag (107). Subsequent vaccination of rabbits with the various chimeric VLP constructs without adjuvant generated antibody responses to the Pr55 gag carrier component. Interestingly, most of the antibody response was specific to the gag insertion site. Despite generating weak neutralizing antibody responses, vaccination of mice with the different non-adjuvanted chimeric VLPs could generate strong MHC class I CTL responses. However, when the recombinant antigen was adsorbed with alum, its potential for CTL induction was drastically reduced or inhibited (107). Further studies in NHPs using two differing HIV-1 virus-like Pr55 gag VLP vaccine constructs without adjuvant revealed that IM vaccination with both types of VLP induced elevated titers of anti-gag antibody responses, but it was Pr55 gag/env VLPs expressing the full gp120 at the outer surface of the particle, which generated substantial anti-Env antibody titers with a neutralizing phenotype (108). Furthermore, both Gag and Env cytotoxic T lymphocyte (CTL) responses were induced (108).

Collectively, the literature presented suggests that despite Alum’s positive influence in some preclinical experiments and in some clinically approved VLP formulations, its inappropriate use can diminish the effects of test vaccines, resulting in ineffectual immune responses. Thus, inclusion of alum as a VLP adjuvant in any preclinical or clinical trial must be scrutinized.

### Pattern Recognition Receptor (PRR) Agonists

The most highly characterized PAMP sensors are the innate immune system PRRs for which several families have been classified. The C-type lectin toll-like receptors (TLR) are the best-defined families of PRRs. They are expressed by a plethora of mammalian cells as either endosomal or plasma membrane bound receptors. Cell surface TLRs (4, 1/2, 2/6, 5) detect conserved microbial patterns present on the cell surface such as LPS and flagellin, while endosomal TLRs (3, 7, 8, 9) detect microbial genomic material such as double-stranded RNA and DNA and single-stranded RNA (109). Detection of bacteria or viral particles by the various TLRs expressed by APCs, such as DCs, macrophages, and B cells, is normally followed by endocytosis/phagocytosis and subsequent processing and presentation to T cells *via* MHC. The MHC presentation to T cells occurs in the context of several signals, including, co-stimulatory molecules, cytokines, and by TLR ligation. Collectively, this stimulates T cells to surpass the minimal activation thresholds for naïve T cells to partake in immune responses. Due to their abilities to activate innate signaling pathways, PRR ligands are an expanding field of study for HIV vaccine design. This includes the use of TLR4 (MPL) and 7/8 (R848) agonists encapsulated in PLGA particles admixed with VLPs expressing SIV gp160 and Gag (110), the incorporation of TLR7/8 (Resiquimod) and 9 (CpG) into oil-in-water emulsions for responses against HIV-1 Env gp140 (111) and the use of TLR3 (Poly IC:LC, Hiltonol), TLR4 (MPL derivative, E6020) and TLR 7 (a proprietary benzonaphthyridine from Novartis) agonists to characterize vaccine-mediated evolution of Env-specific B cell ontogenies (112). Normally, the microbial antigen and TLR ligand are physically associated (i.e., contained within the same pathogenic organism), and therefore are co-delivered to the same phagosomal/endosomal compartment within the APC. During vaccinations, PAMPs are generally admixed with antigens and, therefore, may not be associated as having a common derivation, unless the PAMP and antigen enter the same endosome. The latter requiring large excesses of PAMPs over what is required for APC and T cell activation (113). Therefore, formulating TLR ligands onto the surface or within nano-vaccines serves as a promising way to optimally stimulate APCs. The TLR7 agonist Imiquimod has been approved for topical delivery in humans for genital warts as a 5% cream (Aldara™) (114). However, its systemic application has been limited because of toxicity. Due to its hydrophobic nature, entrapment within polymeric nanoparticles offers an alternative strategy for imiquimod delivery, greatly reducing its toxicity. A study by Jimenez-Sanchez et al., reported on polylactide (PLA)-based micelles with entrapped TLR7 and HIV-1 gag p24 decorating the surface of the nano-system. As would be expected, encapsulated imiquimod induced stronger DC stimulation than free imiquimod with encapsulated ligand triggering NF-κB and MAPK pathways (114). In another study, Francica et al. immunized NHPs with HIV gp140 Env with several adjuvants. When the authors co-formulated gp140 with TLR4/7 agonists and alum, they revealed that anti-Env antibody titers were boosted 3- to 10-fold higher than antibody titers induced with alum and gp140 alone. Interestingly, while TLR4 and alum enhanced a set of inflammatory genes, TLR7 suppressed alum-specific inflammatory genes in preference for interferon-responsive genes (115). This suggests that different TLR ligands can have different transcriptional effects against co-formulated adjuvants.

### MPL and GLA

The detoxified TLR-4 ligand, 3-*O*-deacyl-4′-monophosphoryl lipid A (MPLA), is a lipopolysaccharide (LPS)-derived, heterogeneous blend of varying length MPL molecules from *Salmonella minnesota R595*. The MPLA extraction and treatment procedure resulted in three distinct modifications compared to the parent molecule: (1) the removal of the core polysaccharide containing the O-antigen, (2) the removal of one phosphate group, and (3) one fatty acid chain (116, 117). MPLA has been shown to promote T_H_1 immune responses and to have a favorable safety profile when compared to LPS (118–120). Since the identification of MPLA as an adjuvant, newer generations of TLR-4 agonists have been developed, such as a synthetic lipid A-like molecule called GLA. GLA adjuvant is either administered in aqueous nanosuspension (AF) or more often, co-formulated with a squalene-based oil-in-water nanoemulsion (SE). GLA-SE is now a clinical stage vaccine adjuvant that also promotes T-bet-dependent T_H_1 immune responses and enhances protection against a range of intracellular pathogens (97). In fact, it is unsurprising since most TLRs use MyD88 except TLR3, that GLA-SE has been shown to signal through TRIF and MyD88 and that both are necessary for GLA-SE adjuvant activity (121). CD4^+^ T cells are the central orchestrators of adaptive immune responses and provide the critical instructions for directing antibody production by B cells and CD8 T cell memory development (122, 123). Therefore, it is notable that, in terms of T cell responses, GLA-SE has been shown to promote polyfunctional CD4 T cell reactions characterized by CD154, IFN-g, TNF-a, GM-CSF, and IL-2 expression and the potential for GLA-SE induced IFN-a to trap immune cells in draining lymph nodes *via* CD69 expression (97). Immunization with *Mycobacterium tuberculosis* antigen and GLA-SE has resulted in elevated antigen specific B cell responses, elevated antibody concentrations, and increased follicular T helper and T_H_1 biased CD4 T cell numbers compared with adjuvants alum, GLA without SE, or SE alone (124). Furthermore, there is evidence to suggest that GLA-SE augments early innate IFN-g production by CD8 T and NK cells (97).

More recently, it has become clear that TLR4 agonists and TLR7 agonists may be highly synergistic in amplifying immune responses. This can be seen by increased cytokine secretion, heightened germinal center formation, and both antibody class switching and diversity (125, 126). Hence, the development of a nanoliposome formulation that co-localizes TLR4 and TLR7 agonists to the same APCs, which synergistically enhances immune responses have been developed. These TLR4/7 lipid nanoparticle adjuvants have been co-formulated with VLP containing SIVmac239 Env and Gag with the aim of comparing the immunogenicity of soluble gp140 Env and a protein immunogen to Env expressed on the surface of VLPs (127). Interestingly the nanoparticle adjuvant used in combination with soluble gp140 vaccine antigen performed better than the nanoparticle adjuvant co-formulated with VLPs. Specifically, the anti-Env antibody responses and Env-specific plasmablast numbers were greater in magnitude in serum for the soluble gp140 compared to VLP vaccine groups (127). To test the efficacy of TLR4/7-adjuvanted VLPs compared to soluble gp140, vaccinated macaques were challenged weekly with an SIVsmE660 swarm, and although there was a delay in infection with the adjuvanted VLP treatment group compared to the adjuvanted soluble gp140 treatment group, it was not statistically significant. The inclusion of the adjuvant did, however, enhance protection for both soluble gp140 and VLPs compared to the non-adjuvanted controls (127).

### Adjuvant System 04 (AS04)

Adjuvant System 04 is a new combination adjuvant developed by GlasxoSmithKline Biologicals and consists of Aluminum salts and MPLA. The AS04 adjuvant is utilized in two licensed vaccines, Cervarix^®^ and Fendrix^®^. Cervarix^®^ contains VLPs of the L1 capsid protein from oncogenic HPV strains 16 and 18 expressed through an insect cell expression system. Fendrix^®^ works against hepatitis B virus (128–130). It should be pointed out that a second HPV vaccine based on recombinant expression of major capsid antigen L1 in yeast is marketed by Merck &Co. under the brand name Gardasil^®^(131). In terms of Cervarix^®^, AS04 was selected as the adjuvant of choice because of its safety performance in mice and NHP studies and because it demonstrated induction of higher quality antibody and neutralizing anti-HPV titers response compared to alum alone (99). In the HPV16/18 clinical study, the VLP formulation adjuvanted with AS04 was proven effective against cervical intraepithelial neoplasia lesions and protective against strains phylogenetically related to HPV16/18 such as HPV31, HPV33, and HPV45 (132, 133). Comparing the AS04-adjuvanted HPV16/18 vaccine to alum adjuvanted HPV6/11/16/18 in women aged 18–45 revealed the former to elicit higher levels of neutralizing antibodies and higher frequencies of memory B cells and CD4 T cells (134).

### MF59

The adjuvant MF59 was first approved in Italy in 1997 after Novartis conducted clinical trials in 1992 (135). MF59 is a squalene-based oil-in-water emulsion stabilized by Tween 80 and Span 85 surfactants (136). Due to its extensive safety profile and efficacy in preclinical and human clinical trials, it is now FDA approved and a component in licensed influenza vaccines such as FLUAD^®^, Aflunov^®^, Focetria^®^, and Celtura^®^ (137). In addition, it is also being evaluated as an adjuvant in candidate HIV vaccine formulations. Although a complete understanding behind the mechanism of action of MF59’s is still being defined, the widely held theory is that MF59 activates injection site tissue-resident APCs such as macrophages along with the transient release of ATP from the injected muscle (78). Upon activation, these tissue-resident cells secrete various chemokines and cytokines, which causes a localized inflammatory reaction, resulting in recruitment of more immune cells into the injection site. Vaccine antigen–APC interaction is, therefore, increased, augmenting transport of antigen into local draining lymph nodes and T cell priming. In terms of humoral immune responses, MF59 has been shown to increase antibody affinity maturation to vaccine antigens as well as the diversity of epitopes recognized (138). How this occurs may be due to findings that MF59 enhances antigen-specific IgG antibody responses to vaccine antigens by promoting T follicular helper cell (T_FH_) responses (139). While few studies have evaluated ways to augment the development of T_FH_ cell responses through vaccination, these highly specialized cells are critical for humoral immunity through provision of help by means of both contact-dependent and independent mechanisms with T_FH_ cell responses directly controlling the magnitude of germinal center B cell responses (140). Therefore, MF59 represents a very promising adjuvant for future vaccine development. Recently, Vargas-Inchaustegui, et al. demonstrated that an SIV vaccine-prime consisting of an Adenovirus 5 host range (Ad5hr) mutant, encoding SIV Gag, Nef, Rev, and Env followed by IM boosts with SIV monomeric gp120 or oligomeric gp140 protein adjuvanted with MF59 can induce long-lived germinal center, Env-specific IL-21^+^T_FH_ cells in rhesus macaque lymph nodes (140). However, while MF59 has been reported to promote germinal center formation and T_FH_ cell induction in previous studies (139, 141), within this study the corresponding effects of the boost to one of the components of the vaccine (SIVmac239 Env) were not as pronounced as that seen in Ad5hr vaccine-prime (140). Whether this lack of increased T_FH_ cell induction by MF59 adjuvanted boost is due to nature of the vaccine antigen being used or its exposure time in the body compared to the viral vector remains to be determined (140).

Previously, Guillon et al. quantitatively and qualitatively tested the antibody response in rabbits induced by PLA nanoparticles and MF59 adjuvant. Using three different antigens, HIV-1 p24, WT HIV-1 Tat, and a detoxified Tat, both adjuvant platforms induced similar levels and kinetics of serum IgG (142). Strikingly, the authors noted differences in the antigenic domains that elicited the antibody response. Serum from p24 vaccinated rabbits showed MF59 induced antibodies that recognized peptides all along the p24 protein sequence while the PLA nanoparticles directed the antibody response toward an immunodominant domain of p24 (190–224). Potentially indicating that adsorption of antigens onto the surface of particles might unmask, or alter the conformation of antigens, revealing alternative immunodominant epitopes not revealed by soluble antigens (142).

### ISCOMATRIX

This adjuvant was designed as an improvement over its predecessor, the ISCOM (immunostimulating complex) adjuvant. The original formula was a mix of saponin, cholesterol, phospholipid, and other hydrophobic compounds (143). The major drawback to the original formulation was that the vaccine antigen had to be integrated into the hydrophobic structure of the adjuvant, greatly limiting the types of antigen that could be administered with ISCOM (100). To address these issues, Pearse & Drane developed ISCOMATRIX, which did not require the incorporation of antigen. This newer formulation was similar; however, was produced using purified fractions of *Quillaja Saponaria*, cholesterol, and phospholipids. When formulated together, the lipid and QS21 components assemble into 40–50 nm cage-like structures that entrap the vaccine antigen. Unlike other adjuvant systems, the ISCOMATRIX–antigen preparation infiltrates the draining lymph node and interacts with lymph node-resident DCs and other APCs within the first 2 h of immunization. Interestingly, there are two waves of antigen presentation when ISCOMATRIX is used. The first round of antigen presentation occurs when ISCOMATRIX–antigen complexes infiltrate the draining lymph node, whereas the second round of antigen presentation occurs 24–48 h post-inoculation. This is due to tissue-resident DCs that take up ISCOMATRIX–antigen complexes and traffic to draining lymph nodes.

Morelli et al. describe ISCOMATRIX as an integrated adjuvant system because it combines the innate and adaptive arms of the immune system in a TLR-independent but MyD88-dependent manner. In particular, when DCs take up ISCOMATRIX into endosomes or phagolysosomes, these cellular compartments undergo acidification and releases the ISCOMATRIX–antigen complexes into the cytoplasm of the cell. This enables presentation through MHC class I pathway for cross-presentation to CD8 T cells. As expected, antigen is also presented on MHC class II for presentation to CD4 T cells and induction of B cell responses. In terms of T cell responses toward ISCOMATRIX–antigen complexes, it activates both T_H_1 and T_H_2 responses as noted by rapid IL-5 and IFN-g production following immunization (144).

As mentioned previously, Ingale et al. evaluated the *in vivo* delivery of soluble JRFL SOSIP trimers in ISCOMATRIX and JRFL SOSIP trimer-conjugated liposomes in ISCOMATRIX. Isolation of lymph node cells indicated increased percentages of GL7+ germinal center B cells from mice administered the liposome-conjugated vaccine (58). Within this study, the delivery and evaluation of well-ordered, high-density Env trimers was the main goal of the research, as such there was no evaluation of the SOSIP Env trimers in the absence of ISCOMATRIX, making it difficult to understand the precise effect of the adjuvant. In a separate study, evaluating germinal center formation and neutralizing antibody responses to HIV Env trimers, Havenar-Daughton et al., using a modified BG505 SOSIP (BG505 SOSIP.v5.2), compared two different adjuvants (PLGA/R848 + MPL vs ISCOMATRIX) for generation of autologous tier 2 neutralizing antibody production in NHPs. In this example, approximately 75% of the vaccinated NHPs generated a detectable neutralizing antibody response but neither adjuvant significantly outperformed the other in terms of elicited Env antibody titer, V3 peptide antibody binding titer or neutralizing antibody titer (145). Interestingly, three NHPs from the PLGA group and 1 NHP from the ISCOMATRIX group were identified as top tier 2 autologous neutralizing antibody producers (titers >1:200). Although it should be noted that both groups had different numbers of animals, with the PLGA treatment group having the most. While these results were promising, no viral challenge was performed (145). Collectively, this suggests that ISCOMATRIX is a promising vaccine adjuvant with the potential to significantly augment B cell immune responses.

### Surface Functionalized Nano-Vaccines

The co-delivery of vaccine antigens with adjuvants has long been known to augment both B and T cell immune responses. As previously mentioned, current scientific dogma suggests for optimal adjuvant activity, the vaccine antigen and the adjuvant should be targeted to the same APC. Co-delivery of vaccine antigens and adjuvants can normally be achieved using covalent linkages between the two components, ensuring that the antigen and adjuvant will end up in the same endo-lysosomal compartments for maximal PRR engagement and proteolytic degradation. However, due to the increased costliness and in some cases, difficulty of conjugating certain complex antigens or heavily glycosylated antigens to the adjuvant makes this process unfavorable. Therefore, newer ways to formulate antigens and adjuvants are necessary. Interestingly, the use of nano-vaccines offers an alternative mechanism by which antigens and adjuvants can be co-delivered for optimal immune responses. Through cell surface expression, a range of recombinant vaccine adjuvants can be incorporated into budding VLP nano-vaccines, ensuring that surface expressed immunogens are presented alongside the adjuvant. In a study by Franco et al., immuno-stimulatory murine CD40L was expressed on the surface of HIV VLPs, with the aim of targeting HIV-1 gag proteins to DCs and activating the CD40 receptor (146). Through fusion to the CD40L ectodomain with baculovirus gp64, the authors significantly enhanced CD40L expression by the VLPs and demonstrated that the CD40L/gp160 VLPs enhanced IL-12 production from DCs. Further *in vivo* testing of the CD40L/gp160 VLPs in mice demonstrated the vaccine construct to induce strong CD4 and CD8 cell-mediated immune responses compared to CD40L-VLP constructs expressing either full length murine CD40L or murine CD40L fused to gp41(146).

Numerous studies have shown that cytokines serve to be promising adjuvants for vaccines. For instance, an SIV239-based heterologous prime-boost vaccine, co-expressing GM-CSF, was shown to convey 71% protective efficacy against intrarectal SIV_SM_E660 challenge when compared to 25% in the non-GM-CSF adjuvanted group (147). In this case, the GM-CSF adjuvant was shown to enhance the avidity of the anti-Env IgG antibody response, augment the neutralizing activity of the sera, and enhance ADCC activity (147). More recently, a gain of function fusokine (GM-CSF + IL-4, aka GIFT4) membrane bound construct was utilized as a VLP bearing, B cell adjuvant (148). Anchoring of the fusokine into the Env-enriched VLP membrane was achieved by fusing CD59 glycolipid, glycophosphatidyl-inositol (GPI) sequence to GIFT4. The chimeric VLP constructs were administered as an IM-prime, IN-boost in Guinea pigs resulting in high levels of systemic antibody responses. In addition to this, the elicited antibodies exhibited higher avidity, improved neutralization breadth, and were detected in both serum and mucosal secretions (148).

Flagellin, in the monomeric form, is a TLR5, MyD88-dependent signal agonist derived from Gram-negative bacteria flagella. Flagellin is the major protein of the flagellum filament, which extends from the outer membrane of many locomotive Gram-negative bacteria and is responsible for the organism’s locomotive ability. Flagellin is highly pro-inflammatory, activating NF-kB in a variety of cells, such as epithelia and APC populations such as monocytes and DCs (98). As the TLR5 receptor is found to be expressed in APCs, it has been suggested that flagellin-containing VLPs may be phagocytosed after TLR5 binding and enhance antigen presentation due to innate signaling within the same cell. In an interesting study by Wang et al., a membrane-anchored form of flagellin was incorporated into a chimeric influenza VLP (149). The results clearly demonstrated that flagellin-containing VLPs could elicit significantly higher antibody responses than unadjuvanted VLPs and that the adjuvant activity was not restricted to humoral immunity. In the latter case, MHC class I and II peptide stimulated splenocyte cultures were shown to have increased cellular immune responses and the mice vaccinated with adjuvanted VLP to be more resistant to heterologous influenza challenge infection than non-adjuvanted controls (149). The membrane-anchored flagellin was also combined into HIV VLPs as a molecular adjuvant using the guinea pig model (150). Both systemic (IM) and mucosal (IN) routes of vaccination elicited serological and mucosal antibody responses, with IgG sera levels correlating with neutralization. Of note, the neutralizing antibody response was broadened against the five tested strains from clades B and C (150).

Surface functionalizing of nanoparticles can also increase the bioavailability of vaccine antigen cargos through enhanced vaccine penetration in tissues. For instance, through decorating the surface of nano-vaccines with mucosal M cell targeting moieties, the bioavailability of mucosally applied vaccine formulations can be increased and antigen-specific immune responses significantly augmented. For instance, through surface functionalizing of PEGylated PLGA nano-vaccines with integrin binding RGD tripeptide (Arg-Gly-Asp), Garinot et al. were able to significantly increase the transport of particles using an *in vitro* model of follicle associated epithelium (Caco-2 cells and Raji B cells) and also monocultures of Caco-2 cells. Oral application of the RGD-nanoparticles was also shown to be immunogenic in some mice, with some animals having elevated anti-OVA IgG antibody responses and splenic IFN-γ T cell responses (151). Additional M cell targeting moieties, such as fucose binding lectin *Ulex euopaeus* agglutinin1 and reovirus sigma protein-1 have also previously been studied as virus, nanoparticle and microparticle targeting strategies for drugs and vaccines (152–156). Alternatively, cell penetrating peptides such as HIV-1 Tat, or CD71 receptor targeting protein such as transferrin have been bio-coupled to vaccine antigens and nano-vaccines for delivery to various cell types and organs (157). Therefore, the functionalization of the surface of nano-vaccines represents an exciting area of active investigation with the potential to significantly tailor vaccines to specific cells, tissues, and organs for optimal immune protection.

## Summary and Conclusion

The last three decades has seen tremendous advancements in our understanding of HIV acquisition, treatment, and prevention. During this time, several vaccine efficacy trials have been completed and all have failed to prevent HIV infections apart from the modestly effective RV144. A major impediment to a successful vaccine has been antigen design and delivery. Crucial to overcoming this roadblock has been the development of nanoparticles to stabilize and orientate candidate vaccine immunogens for optimal immune triggering. Within this review we have covered the basis for vaccine development and the promising nanoparticle-based approaches to answer some of the very complicated issues surrounding HIV vaccine progress. Nanoparticle-based vaccines have demonstrated themselves to be versatile, and depending on which antigen, adjuvant and delivery route used, can have significant impacts on prevailing immune responses. While nanoparticle vaccines hold great promise, a significant number of unknowns still exist, and when answered could greatly advance our understanding on how to maximally deliver promising vaccine immunogens. These include a better understanding of the effects of charge, size, structure, and potential toxic side effects with various nanoparticle platforms.

While the major focus of this nanoparticle review has been on the development of neutralizing antibody, nanoparticle formulations targeting the V1/V2 region of Env by non-neutralizing antibody is a significant area of interest and needs to be explored further. Justification for this is drawn from the correlates of protection from the RV144 study, which is being further evaluated and validated by on-going clinical trials in Thailand and Africa. In addition, although most vaccines are delivered systemically, HIV is predominantly transmitted through mucosal surfaces and as such, it is imperative that vaccine candidates be developed that can either augment mucosal immunity or be designed to draw protective systemic responses into the mucosal environment. The latter point would enable the provision of protective frontline responses within tissues at risk of infection. The only caveat being that mucosal tissue activation and vaccine-induced inflammation be carefully managed, to not increase the likelihood of transmission by growing the number of potential target cells.

Finally, the use of nanoparticle technology will also undoubtedly have a significant role to play in therapeutic HIV vaccines. The thought of an HIV-1 cure was once regarded a dream. Now, growing evidence suggests that a cure could be a reality and the generation of vaccine induced antiviral immunity will take center stage. There are numerous cure technologies that currently being evaluated, however it is the “Shock and Kill” tactic that has garnered significant attention. Several latency reversal agents have been devised to reactivate (shock) latent virus into transcriptional activity; however, vaccines that can prime for protective antibody and cytotoxic T and NK cell responses will be essential for subsequent viral eradication (kill).

We are clearly entering an exciting time for HIV vaccine development and nanoparticle formulations will have a lot to offer. As long as there is continued support for the nanoparticle vaccine effort, prophylactic and therapeutic HIV/AIDS interventions will continue to advance until such time as a vaccine becomes a reality.

## Authors Contributions

YG, CW, and JM wrote sections of the manuscript and YG, CW, and JM edited the manuscript.

## Conflict of Interest Statement

The authors declare that the research was conducted in the absence of any commercial or financial relationships that could be construed as a potential conflict of interest.
